# Ghrelin Gene Variants Influence on Metabolic Syndrome Components in Aged Spanish Population

**DOI:** 10.1371/journal.pone.0136931

**Published:** 2015-09-16

**Authors:** Mireia Mora, Victoria Adam, Elisabet Palomera, Sebastian Blesa, Gonzalo Díaz, Xavier Buquet, Mateu Serra-Prat, Juan Carlos Martín-Escudero, Ana Palanca, Javier Felipe Chaves, Manuel Puig-Domingo, The Mataró Aging Study Group

**Affiliations:** 1 Department of Endocrinology and Nutrition, Hospital Clínic of Barcelona, Barcelona, Spain; 2 Genotyping and Genetic Diagnosis Unit, Research Foundation, Hospital Clinic of Valencia INCLIVA, Valencia, Spain; 3 Research Unit and *Ciberhep*, Hospital of Mataró, Mataró, Spain; 4 Hospital Clínico of Valladolid, Valladolid, Spain; 5 Biochemistry Service, Hospital of Mataró, Mataró, Spain; 6 Internal Medicine Department, Hospital Río Hortega, University of Valladolid, Valladolid, Spain; 7 Department of Endocrinology and Nutrition, Hospital Universitari Germans Trias i Pujol, Badalona, Spain; 8 CIBER Diabetes and Associated Metabolic Diseases (CIBERDEM), Barcelona, Spain; 9 Centro de Investigación Biomédica en Enfermedades Raras (CIBER-ER), Madrid, Spain; Harvard Medical School, UNITED STATES

## Abstract

**Background:**

The role of genetic variations within the ghrelin gene on cardiometabolic profile and nutritional status is still not clear in humans, particularly in elderly people.

**Objectives:**

We investigated six SNPs of the ghrelin gene and their relationship with metabolic syndrome (MS) components.

**Subjects and Methods:**

824 subjects (413 men/411 women, age 77.31±5.04) participating in the Mataró aging study (n = 310) and the Hortega study (n = 514) were analyzed. Anthropometric variables, ghrelin, lipids, glucose and blood pressure levels were measured, and distribution of SNPs -994CT (rs26312), -604GA (rs27647), -501AC (rs26802), R51Q (rs34911341), M72L (rs696217) and L90G (rs4684677) of the ghrelin gene evaluated. Genotypes were determined by multiplex PCR and SNaPshot minisequencing. MS (IDF criteria) was found in 54.9%.

**Results:**

No association between any of the SNPs and levels of total fasting circulating ghrelin levels was found. C/A-A/A genotype of M72L was associated with increased risk of central obesity according to IDF criteria, while G/A-G/G genotypes of -604GA with reduced risk. A/A genotype of -501AC polymorphism was associated to decreased BMI. In relation to lipid profile, the same genotypes of -604GA were associated with increased total cholesterol and LDL-cholesterol and -501AC with reduced triglycerides. There were no associations with systolic or diastolic blood pressure levels or with hypertension, glucose levels or diabetes and ghrelin polymorphisms. However, G/G genotype of -604GA was associated with glucose >100 mg/dL. Haplotype analysis showed that only one haplotype is associated with increased risk of waist circumference and central obesity. The analysis of subjects by gender showed an important and different association of these polymorphisms regarding MS parameters.

**Conclusion:**

Ghrelin gene variants -604GA, -501AC and M72L are associated with certain components of MS, in particular to BMI and lipid profile in elderly Spanish subjects.

## Introduction

Ghrelin is an hormone secreted especially in the stomach with an orexigenic effect, stimulating appetite and food intake and playing an important role in regulating the body energy homeostasis [1,2]. It has been suggested that age-related decline in circulating ghrelin concentrations is one of the potential mechanisms involved in reducing appetite in the elderly [3,4]. However, this decrease in fasting ghrelin concentration in people over 70 years is quite modest and its physiological relevance remains unclear [5]. An active participation of ghrelin in the regulation of energy homeostasis probably persists until advanced age, as different studies have shown greater ghrelin circulating concentrations as a potential compensatory mechanism in malnourished aged subjects in comparison to well-nourished individuals [6,7]. Ghrelin also appears to have vascular protective actions, and thus, its role in the development of different cardiometabolic disturbances has been proposed.

The role of ghrelin in the metabolic syndrome (MS) risk is controversial, especially when we take into account the different two major isoforms of ghrelin (desacyl and acylated ghrelin) [8]. Circulating ghrelin has been associated to the metabolism of glucose and lipids, considering that both forms seem to have opposite effects. Acyl-ghrelin reduces the release of insulin depending on glucose, decreases the sensibility to insulin and promotes hyperglycemia while desacyl-ghrelin increases the sensibility to insulin and maintains euglycemia, suggesting the opposite effect of the two isoforms of ghrelin in the hepatic metabolism of glucose [9]. Moreover, ghrelin has a lipogenic effect, inhibiting the synthesis of fatty acids [9]. In relation to MS, OPERA study has shown a negative correlation between levels of total ghrelin and the number of components of MS and MS per se: levels of ghrelin decrease when the number of components of MS increases [10]. Previous data of the Mataró Aging Study have also shown this association [11]. Recent reports have found that, as the number of components of the metabolic syndrome increase, a progressive reduction in circulating desacyl ghrelin is observed, while conversely, acylated ghrelin levels are increased [12] or unchanged [13–14] in parallel with the presence of components of MS. However, it is not clear if this association is causative or not. Moreover, the role of ghrelin in the development of MS in aging individuals is still not well known.

The ghrelin polymorphisms most studied to date are located in the promoter and in the coding regions of the gene and some of them have implications for gene activity [15]. The best known SNPS are Arg51Gln, located in the area that regulates the generation of the active mature hormone, and Leu72Met and Gln90Leu, located elsewhere in the gene [16]. Leu72Met (C247A) is in the gene region located between the coding regions of obestatin and mature ghrelin. The functional consequences of this variation are unknown, because even if the sequence of the mature ghrelin does not change, changes in mRNA stability or changes in the processing of the protein could alter the secretion of ghrelin and / or its activity [17]. The SNP for Gln90Leu (A265T) causes a change in an amino acid peptide obestatin and the possible consequences of this change are unknown [18]. The association of ghrelin polymorphisms with MS has been previously studied with no coincident results: some show no association with metabolic alterations [19] while others have found a relation [16,20,21]. For example, M72L has been associated to MS, high-density lipoprotein, high triglyceride levels and high fasting glucose [16,21]. Individuals carrying the 51Gln allele have a lower prevalence of MS in comparison to non-carriers [16]. The purpose of our study was to describe the distribution of -994CT (rs26312), -604GA (rs27647), -501AC (rs26802), R51Q (rs34911341), M72L (rs696217) and L90G (rs4684677) ghrelin gene polymorphisms and their relationship with MS in an elderly Spanish population.

## Subjects and Methods

### Study population

All subjects of the present study were participants of the Mataró Aging Study (n = 310) and Hortega Study in Valladolid (n = 514), two Spanish population-based cohort studies designed to identify cardiovascular risk factors for frailty and successful aging condition among old people. Table 1 shows the characteristics of both cohorts.

**Table 1:** Characteristics of the individuals by population.

	Whole sample	Mataró (n = 310)	Valladolid (n = 514)	p	Men	Women	p
Age (years)	77.31±5.04	76.99±5.92	77.49±4.43	0.201	77.24±4.85	77.38±5.22	0.684
BMI (kg/m^2^)	28.01±4.19	28.10±4.14	27.94±4.22	0.590	27.28±3.56	28.73±4.63	<0.001
Systolic BP (mmHg)	144.91±22.52	142.25±23.02	146.49±22.10	0.009	142.94±21.10	146.92±23.75	0.011
Diastolic BP(mmHg)	80.94±11.99	80.55±12.62	81.17±11.60	0.479	80.13±11.87	81.76±12.07	0.053
Hypertension n (%)	593 (72.7)	202 (66.4)	391 (76.4)	0.002	289 (70.1)	304 (75.2)	0.102
TC (mg/dl)	209.00±37.80	210.68±37.85	208.02±7.81	0.338	200.19±35.92	217.84±37.63	<0.001
Triglycerides (mg/dL)	169.37±97.95	128.07±75.11	192.95±101.66	<0.001	177.16±104.90	161.56±9.91	0.024
LDL-cholesterol (mg/dL)	123.07±34.30	129.50±33.94	119.38±34.02	<0.001	116.54±33.67	129.61±33.72	<0.001
HD-cholesterol (mg/dL)	51.78±13.09	54.79±12.71	50.05±13.17	<0.001	47.95±11.72	55.61±13.48	<0.001
Glucose (mg/dL)	101.85±25.85	107.33±27.69	98.770±24.26	<0.001	102.89±27.70	100.80±23.84	0.252
Diabetes n (%)	114 (13.9)	65 (21.2)	49 (9.6)	<0.001	56 (13.6)	58 (14.2)	0.796
Waist perimeter (cm)	98.35±11.16	101.93±9.96	96.15±11.29	<0.001	100.80±9.49	95.80±12.15	<0.001
Central obesity (IDF criteria) n (%)	676 (84.1)	282 (92.5)	394 (79.0)	<0.001	320 (89.9)	356 (89.9)	<0.001
MS n (%)	585 (74.1)	213 (74.0)	372 (74.3)	0.928	84 (54.9)	98 (61.0)	0.293

Values are mean ± standard deviation TC: total cholesterol; BMI: body mass index, BP: blood pressure; MS: metabolic syndrome by IDF criteria.

In the Mataró Aging Study, the participants were selected from the inhabitants of Mataró and Argentona (Barcelona, Spain) and have been previously described elsewhere [22]. All non-institutionalized residents aged 70 years or older were eligible for the study. Exclusion criteria included severe physical or mental disability that did not allow visiting the study center and individuals with previous gastric surgery. Sample selection was done on a random basis from the municipal census. A total of 824 individuals of both sexes were invited to participate, this was first done by postal contact, followed by a telephone call from May 2002 to June 2003. Of those invited to participate, 176 (21.3%) were excluded because of non-fulfillment of selection criteria and 87 (10.6%) because of the impossibility of contacting them after three attempts by telephone. Of the remaining 561 individuals, 139 (24.8%) did not accept to participate, 62 (11%) accepted but did not come to the appointment-visit and 47 (8.4%) declined to participate for other non-medical reasons. Finally, 310 cases participated in the study. As more than 90% of the population over 70 in this geographical area of the Maresme in Barcelona are followed at the Primary Health Care Centers of the Maresme Health Consortium, comparisons between individuals initially contacted but not participating in the study and participants performed by using the electronic clinical records database did not show differences in age and sex distribution, or in comorbidities (including cardiovascular, rheumatologic, metabolic and mental disturbances and neoplastic diseases).

Biological samples for genetic studies were obtained from 292 individuals (144 men and 148 women). The Ethics Committee of the Maresme Health Consortium approved the study protocol and all subjects signed an informed consent before entering.

The Hortega Study is a two-stage population-based survey carried out from 1997 to 2003 in adults 14–84 years old from Valladolid (Spain) [23]. The selection of individuals was conducted based on a listing from different local registers, including the universal health care system, which provides a more extensive representation of individuals living in the area than the official census of inhabitants. In the first stage, the 20% of the 214,445 individuals included in the registers was randomly selected and invited by telephone and mail to participate in the study. The response rate was 50%. An independent study was performed to compare participants who did and did not respond, without significant differences in socioeconomic and cardiovascular risk factors. Among the individuals who responded, ~250 individuals were randomly selected from 6 sex-specific age strata to undergo interview and clinical examination and to provide biological samples. If the contact could not be established, or the individual refused to participate in the study, the investigator had the possibility of recruiting another individual from the same strata using a reserve list. Individuals were excluded from this survey for the presence of serious concomitant diseases or disorders that could influence the collection of reliable information, or any mental or social condition that could complicate or prevent participation of the individual in the study. After signing an informed consent form, plasma samples were drawn and stored for metals determination, resulting in 1,502 participants available for the present study. From them, 514 participants were 70 years old or over and included in the present study. The research protocol was approved by the local Ethics Committee of the Hospital Río Hortega.

### Data collection

Questionnaires for geriatric evaluation and physical assessment were performed by trained teams in Mataró and Valladolid with a low inter-team variability regarding all the procedures related to the study. The study protocol included a revision of the electronic medical history of the individual as well as a global geriatric evaluation when individuals entered the study. Chronic and previous diseases, life style factors, education, a physical examination and a laboratory study for biochemical, hormonal and genetic determinations were recorded in a specific database designed for the study. The physical examination included measurement of weight (in kg) and height (in cm); waist circumference was measured in standing position in a line between the last rib and the iliac crest. Body mass index (BMI) was calculated as weight divided by height (meters) squared. Blood pressure was measured twice with the subject seated after 5 minutes rest, the mean of two reading was used in the analyses. These data have been previously described elsewhere [24].

### Hormonal measurements

Blood samples for all measurements were obtained after a 12-hour fast through the night. Glucose and lipids were analyzed by enzymatic techniques. Total plasma ghrelin concentrations were measured with a human radioimmunoassay (RIA) kit (Linco Research Inc, St Charles, MO). The detection limit was 93 pg/mL with intra- and inter-assay variation coefficients of 11.1% and 14.7%, respectively. These data have been previously described elsewhere [24].

### Metabolic syndrome (MS) definition

We used the International Diabetes Federation (IDF) definition of MS [25]. Individuals were classified as having MS if the waist circumference was > 94 cm in men or > 80 cm in women plus two or more of the following: (i) arterial blood pressure > 130/85 mmHg or antihypertensive treatment, (ii) triglycerides > 150 mg/dL or use treatment, (iii) high density lipoprotein (HDL) ≤ 40 mg/dL in men or ≤ 50 mg/dL in women, or (iv) fasting glucose ≥ 100 mg/dL or previous diabetes diagnosis. Additionally, for data analysis in relation to ghrelin polymorphisms, waist circumference was also used as a continuous variable.

### Ghrelin gene polymorphisms

Six single-nucleotide polymorphisms (SNPs) of the ghrelin gene (*GHRL*) were investigated in this cohort: -994CT (rs26312), -604GA (rs27647), -501AC (rs26802), R51Q (rs34911341), M72L (rs696217) and L90G (rs4684677). DNA was isolated from peripheral blood cells using the Chemagic System (Chemagen; Baesweiler, Germany). Polymerase chain reaction (PCR) amplicons were designed by Primer3 program [26], to amplify the regions of interest of promoter, exon 1, exon 3 and exon 4 of *GHRL*. The size of PCR products was analyzed by electrophoresis on 2% agarose gels. Products were treated with Exonuclease I (Amersham Biosciences) and shrimp alkaline phosphatase (Amersham Biosciences) to remove excess primers and deoxynucleotide triphosphates. For the examination of the six SNPs, extension SNaPshot primers specific to the polymorphic sites were used for the SNaPshot minisequencing reaction using the ABI PRISM SNaPshot Multiplex Kit (Applied Biosystems). The resulting products were purified by using one unit of Calf Intestine Phosphatase (New England Biolabs, Ipswich, MA). Snapshot products were resuspended in 4.5 μL Hi-Di™ Formamide (Applied Biosystems) and 0.5 μL GeneScan Size Standard. They were then electrophoretically analyzed using a DNA Analyzer 3730 (Applied Biosystems). The results of genotyping were analyzed and evaluated by GeneMapper software v. 3.7 (Applied Biosystems). All polymorphisms were in Hardy-Weinberg equilibrium. These data have been previously described elsewhere [24].

### Data analysis

Categorical variables were expressed by percentage and continuous data by mean (standard deviation). Allele and genotype frequencies and Hardy–Weinberg equilibrium for every SNP were calculated by chi-square test with one degree of freedom. SNPs associations with MS components were analyzed by ANOVA test for data with a normal distribution and the Kruskal-Wallis test for data without a normal distribution. Analysis of association with a response variable was based on linear or logistic regression depending on quantitative or categorical variables. The analyses were performed as crude and then adjusted for age, gender and BMI. We started with a co-dominant model of inheritance in the association studies and if the mean of two genotypes (one homozygous and heterozygous) were similar, we used dominant or recessive models. The model with lower AIC (Akaike information) was used for every SNP. Haplotypes were estimated by the Expectation Maximization Algorithm, and haplotype association with different components of MS were calculated by logistic regression models and adjusted for age, gender and BMI as covariates. All analyses were performed using SPSS package and SNP Stats software [27]. We considered results to be statistically significant when association showed a *P* value <0.05. Odds ratio was used to evaluate the association of the different pathological conditions considered with each SNP or haplotype. These data have been previously related [24].

## Results

### Distribution of ghrelin gene polymorphisms in relation to gender and correlation with ghrelin levels

Eight hundred and twenty-four individuals were included in the present study (413 men/411 women; 50.1% vs 49.9%, respectively); their anthropometric characteristics are described in Table 1. In relation to components of MS according to IDF criteria, 84.1% of individuals had high waist perimeter (central obesity-COB-), 45.2% had triglyceride levels higher than 150 mg/dl, 39.0% had low HDL levels, 72.7% had hypertension and 13.9% were diabetic; as a consequence, 74.1% had MS.

The prevalence of the different genotypes (-994CT, -604GA, -501AC, R51Q, M72L and L90G) is shown in **S1 Table**. No differences were observed between genders in the distribution of polymorphisms. Linkage disequilibrium of the polymorphisms is shown in **S2 Table**.

### Ghrelin gene polymorphisms, ghrelin levels and components of metabolic syndrome

No associations were found between the six SNPs and the levels of total fasting circulating Ghrelin levels in the subgroup of individuals in which this was evaluated (Mataró cohort).

According to IDF criteria, M72L and -604GA polymorphisms were associated with COB; these associations remained statistically significant after BMI, age and gender adjustment (Table 2). Haplotype analysis showed a significant association of haplotype 4 with increased waist perimeter and central obesity risk, which also remained significant after BMI, age and sex adjustments (Table 3) and including the two alleles associated with increased risk for COB found in.

**Table 2:** Ghrelin polymorphisms and their association with waist, central obesity, BMI and obesity as defined by IDF.

**SNPs**	**Model**	**Genotype (N)**	**Waist (cm) Mean (SD)**	**Difference (95% CI) (cm)**	**P-value**	**P-value adjusted** *	**CENTRAL OBESITY COB (%)**	**CENTRAL OBESITY No COB (%)**	**OR (95% CI)**	**P-value**	**P-value adjusted** *
-994CT rs26312	Dominant	C/C (n = 620)	98.47 (0.44)	0.00			518 (79)	102 (81.6)	1.00		
-994CT rs26312	Dominant	C/T- T/T (n = 161)	97.51 (0.94)	-0.96 (-2.90–0.97)	0.33	0.36	138 (21)	23 (18.4)	1.18 (0.72–1.93)	0.50	0.52
-604GA rs27647	Dominant	A/A (n = 237)	98.89 (0.74)	0.00			207 (31.6)	30 (23.8)	1.00		
-604GA rs27647	Dominant	G/A- G/G (n = 543)	97.94 (0.48)	-0.94 (-2.66–0.77)	0.28	0.19	447 (68.3)	96 (76.2)	0.67 (0.43–0.05) **0.57(0.33–1.00)** *	0.074	**0.045**
-501AC rs26802	Dominant	A/A- (n = 311)	98.37 (0.04)	0.00			256 (39.1)	56 (44.4)	1.00		
-501AC rs26802	Dominant	A/C-C/C (n = 468)	98.14 (0.52)	-0.23 (-1.84–1.38) -0.42 (-1.53–0.70)	0.78	0.46	398 (60.9)	70 (55.6)	1.24 (0.85–1.83)	0.27	0.78
R51Q	-	G/G (n = 784)	98.17 (0.41)	0.00			635 (98.8)	125 (99.2)	1.00		
R51Q	-	G/A (n = 9)	98.67 (4.26)	0.50 (-6.88–7.88)	0.89	0.93	8 (1.2)	1 (0.8)	1.57 (0.20–12.70)	0.65	0.91
M72L rs696217	Dominant	C/C (n = 654)	98.13 (0.46)	0.00			538 (83.5)	116 (92.1)	1.00		
M72L rs696217	Dominant	C/A-A/A (n = 116)	98.52 (1.03)	0.39 (-1.83–2.61)	0.73	0.75	106 (16.5)	10 (7.9)	**2.29 (1.16–4.50) 2.97(1.31–6.73)** *	**0.009**	**0.005**
L90G rs4684677	-	A/A (n = 724)	98.39 (0.41)	0.00			611 (92.9)	113 (90.4)	1.00		
L90G rs4684677	-	A/T (n = 59)	96.95 (1.74)	-1.44 (-4.41–1.53)	0.34	0.16	47 (7.1)	12 (9.6)	0.72 (0.37–1.41)	0.35	0.22
**SNPs**	**Model**	**Genotype (N)**	**BMI (Kg/m2) Mean (SD)**	**Difference (95% CI) (cm)**	**P-value**	**P-value adjusted** *	**OBESITY OB (%)**	**OBESITY No OB (%)**	**OR (95% CI)**	**P-value**	**P-value adjusted** *
-994CT rs26312	Dominant	C/C (n = 608)	28.02 (0.17)	0.00			209 (80.7)	415 (78.6)	1.00		
-994CT rs26312	Dominant	C/T- T/T (n = 163)	27.91 (0.33)	—0.11 (-0.83–0.62)	0.77	0.72	50 (19.3)	113 (21.4)	0.88 (0.61–1.27)	0.49	0.49
-604GA rs27647	Dominant	A/A (n = 232)	28.17 (0.74)	0.00			77 (30.0)	161 (30.5)	1.00		
-604GA rs27647	Dominant	G/A- G/G (n = 538)	27.87 (0.18)	-0.30 (-0.94–0.34)	0.36	0.35	180 (70.0)	367 (69.5)	1.03 (0.74–1.42)	0.88	1
-501AC rs26802	Dominant	A/A (n = 312)	27.58 (0.23)	0.00			219 (41.6)	97 (37.6)	1.00		
-501AC rs26802	Dominant	A/C-C/C (n = 458)	28.24 (0.20)	**0.66 (0.06–1.26)** 0.54 (-0.05–1.14)#	**0.031**	0.076	308 (58.4)	161(62.4)	1.18 (0.87–1.60)	0.29	0.43
R51Q	-	G/G (n = 750)	27.98 (0.15)	0.00			248 (98.0)	517 (99.2)	1.00		
R51Q	-	G/A (n = 9)	27.88 (0.91)	-0.08 (-2.81–2.65)	0.96	1	5 (2.0)	4 (0.8)	2.61 (0.69–9.79)	0.16	0.13
M72L rs696217	Dominant	C/C (n = 644)	27.93 (0.16)	0.00			216 (85.4)	443 (84.9)	1.00		
M72L rs696217	Dominant	C/A-AA (n = 116)	28.08 (0.40)	0.15 (-0.67–0.97)	0.72	0.86	37 (14.65)	79 (15.1)	0.96 (0.63–1.47)	0.85	0.77
L90G rs4684677	-	A/A (n = 716)	27.96 (0.16)	0.00			238 (91.9)	492 (93.0)	1.00		
L90G rs4684677	-	A/T (n = 57)	28.24 (0.56)	0.28 (-0.85–1.40)	0.63	0.64	21 (8.1)	37 (7.0)	1.17 (0.67–2.05)	0.58	0.50

BMI: body mass index. COB: Central obesity; OB: obesity.

*Adjusted for age, gender and BMI.

#Adjusted for age and gender.

N: number; SD: standard deviation.

**Table 3:** Haplotype association in relation to waist, BMI, central obesity and obesity.

								WAIST		COB		BMI		OB	
	-994CT	-604AG	-501AC	R51Q	M72L	L90Q	Frequency	Difference (95% CI)	P-value	OR (95% CI)	P-value	OR (95% CI)	P-value	OR (95% CI)	P-value
1	C	G	A	G	C	A	0.3846	0.00	—-	1.00	—-	0.00	—-	1.00	—-
2	C	A	C	G	C	A	0.2879	0.68 (-0.32–1.69)	0.18	1.12 (0.72–1.75)	0.62	0.45 (-0.08–0.99)	0.098	1.24 (0.87–1.77)	0.24
3	C	A	A	G	C	A	0.1400	0.75 (-0.51–2.01)	0.24	1.59 (0.90–2.81)	0.11	0.09 (-0.59–0.77)	0.8	1.33 (0.83–2.11)	0.23
4	T	A	A	G	A	A	0.0526	2.08 (0.16–4.00)	**0.034**	3.42 (1.26–9.31)	**0.016**	0.01 (-1.06–1.08)	0.98	2.33 (0.98–5.50)	**0.055**
5	C	A	A	G	C	T	0.0301	-1.50 (-3.91–0.91)	0.22	0.66 (0.23–1.87)	0.44	0.71 (-0.6–2.02)	0.29	0.76 (0.34–1.68)	0.5
6	C	G	C	G	C	A	0.0247	2.45 (-0.52–5.41)	0.11	1.84 (0.44–7.72)	0.4	-0.97 (-2.59–0.66)	0.24	0.92 (0.32–2.65)	0.88
7	T	A	C	G	A	A	0.0173	-3.05 (-6.81–0.71)	0.11	3.52 (0.35–34.98)	0.28	0.60 (-1.33–2.53)	0.54	3.29 (0.51–21.39)	0.21
8	T	A	A	G	C	A	0.0142	-0.27 (-4.39–3.85)	0.9	1.55 (0.21–11.39)	0.67	-0.5 (-2.48–1.48)	0.62	1.12 (0.28–4.55)	0.87
9	T	A	C	G	C	A	0.0117	-2.58 (-6.80–1.64)	0.23	0.67 (0.08–5.33)	0.71	1.41 (-1.04–3.86)	0.26	1.61 (0.32–8.18)	0.56
rare	-	-	-	-	-	-	0.0370	-0.60 (-3.26–2.07)	0.66	0.48 (0.18–1.27)	0.14	-0.15 (-1.54–1.24)	0.83	0.74 (0.32–1.70)	0.48

COB: Central obesity; BMI: body mass index; OB: obesity. Waist and COB data have been adjusted for age, sex and BMI; BMI and OB data have been adjusted for age and sex.

Polymorphism -501AC was associated with BMI (p = 0.031), the A/A genotype was associated with lower BMI, showing a trend to significance after adjusting for age and gender (p = 0.076; see Table 2). No associations were found for any of the SNPs studied with obesity. However, when being overweight together with obesity were considered as a single categorical variable (BMI ≥25 Kg/m^2^), R51Q G/G was significantly associated with being overweight in comparison to A/G (OR 4.35 (0.90–20); p = 0.041 after adjusting for age and gender). There were no significant associations of ghrelin haplotypes with BMI or obesity, although haplotype 4 showed a trend for increased obesity risk after adjustment for age and sex (Table 3).

There were no associations of ghrelin polymorphisms with systolic or diastolic blood pressure levels, hypertension, glucose levels or hyperglycaemia, according to IDF criteria, and no association with diabetes.

In relation to lipid profile; -604GA polymorphism was associated with total cholesterol and LDL-cholesterol and -501AC was linked to LDL-cholesterol and triglycerides, but no association was found with HDL-cholesterol (Table 4). For -604GA polymorphism, the A/A genotype showed lower total and LDL-cholesterol but statistical significance was lost after adjustment. According to IDF criteria for MS, as expected, an association was found between triglycerides and -501AC polymorphism: 63.4% carriers A/C+C/C vs 36.6% A/A had pathological levels of triglycerides (OR A/A+C/C vs A/A 1.33 (1.10–1.77); p = 0.041 and 1.35 (1.00–1.82) p = 0.048, after adjusting for age and gender). No association was found with HDL-cholesterol. Haplotype analysis showed that minority haplotypes were associated with variations in triglycerides but not with a risk of having high levels, according to IDF criteria.

**Table 4:** Ghrelin polymorphisms association with total cholesterol, LDL-cholesterol, HDL-cholesterol and triglycerides.

**SNPs**	**Model**	**Genotype (N)**	**TC (mg/dL) Mean (SD)**	**Difference (95% CI) (mg/dL)**	**P-value**	**P-value adjusted** *	**Genotype (N)**	**LDL-C (mg/dL) Mean (SD)**	**Difference (95% CI) (mg/dL)**	**P-value**	**P-value adjusted** *
-994CT rs26312	Dominant	C/C (n = 632)	208.40 (1.50)	0.00			C/C (n = 632)	122.42 (1.36)	0.00		
-994CT rs26312	Dominant	C/T- T/T (n = 162)	211.22 (3.06)	2.82 (-3.74–9.37)	0.40	0.20	C/T- T/T (n = 162)	125.33 (2.76)	2.91 (-3.03–8.84)	0.34	0.15
-604GA rs27647	Dominant	A/A (n = 243)	204.72 (2.25)	0.00			A/A (n = 243)	119.45 (2.07)	0.00		
-604GA rs27647	Dominant	G/A- G/G (n = 550)	210.25 (1.43)	6.14 (0.42–11.86)	0.036	0.42	G/A- G/G (n = 550)	124.65 (1.50)	5.19 (0.02–10.37)	**0.05**	0.24
-501AC rs26802	Dominant	A/A (n = 320)	210.80 (2.18)	0.00			A/A (n = 320)	126.11 (2.00)	0.00		
-501AC rs26802	Dominant	A/C-C/C (n = 473)	207.87 (1.71)	-2.93 (-8.31–2.46)	0.29	0.59	A/C-C/C (n = 473)	121.13 (1.53)	-4.98 (-9.85–-0.11)	0.045	0.21
R51Q	-	G/G (n = 773)	208.88 (1.37)	0.00			G/G (n = 773)	123.15 (1.24)	0.00		
R51Q	-	G/A (n = 9)	200.11 (7.75)	-8.77 (-33.68–16.14)	0.49	0.47	G/A (n = 9)	109.22 (7.28)	-13.93 (-36.52–8.67)	0.23	0.34
M72L rs696217	Dominant	C/C (n = 667)	208.47 (1.45)	0.00			C/C (n = 667)	122.65 (1.31)	0.00		
M72L rs696217	Dominant	C/A-A/A (n = 116)	200.11 (7.75)	2.03 (-5.44–9.50)	0.59	1	C/A-A/A (n = 116)	124.82 (3.48)	2.17 (-4.61–8.95)	0.74	0.73
L90G rs4684677	-	A/A (n = 737)	208.56 (1.40)	0.00			A/A (n = 737)	122.55 (1.26)	0.00		
L90G rs4684677	-	A/T (n = 59)	212.88 (4.64)	4.32 (-5.71–14.35)	0.40	0.65	A/T (n = 59)	127.66 (4.35)	5.12 (-3.97–14.20)	0.27	0.40
**SNPs**	**Model**	**Genotype (N)**	**HDL-C (mg/dL) Mean (SD)**	**Difference (95% CI) (mg/dL)**	**P-value**	**P-value adjusted** *	**Genotype (N)**	**TG (mg/dL) Mean (SD)**	**Difference (95% CI) (mg/dL)**	**P-value**	**P-value adjusted** *
-994CT rs26312	Dominant	C/C (n = 632)	51.66 (0.52)	0.00			C/C (n = 632)	169.67 (3.99)	0.00		
-994CT rs26312	Dominant	C/T- T/T (n = 162)	52.68 (1.09)	1.02 (-1.25–3.29)	0.38	0.42	C/T- T/T (n = 162)	166.48 (6.88)	-3.19 (-20.10–13.71)	0.71	0.80
-604GA rs27647	Dominant	A/A (n = 243)	51.92(0.84)	0.00			A/A (n = 243)	167.09 (6.49)	0.00		
-604GA rs27647	Dominant	G/A- G/G (n = 550)	51.78 (0.56)	-0.14 (-2.12–1.84)	0.89	0.55	G/A- G/G (n = 550)	169.92 (4.12)	2.83 (-11.97–17.63)	0.71	0.85
-501AC rs26802	Dominant	A/A (n = 320)	52.11 (0.76)	0.00			A/A (n = 320)	161.68 (5.29)	0.00		
-501AC rs26802	Dominant	A/C-C/C (n = 473)	51.60 (0.59)	-0.50 (-2.36–1.36)	0.60	0.73	A/C-C/C (n = 473)	174.17 (4.59)	**12.49 (-1.38–26.37) 18.05 (4.55–31.55)** *	**0.07**	**0.03**
R51Q	-	G/G (n = 773)	51.87 (0.47)	0.00			G/G (n = 773)	167.87 (3.51)	0.00		
R51Q	-	G/A (n = 9)	52.78 (4.13)	0.91 (-7.72–9.55)	0.84	0.51	G/A (n = 9)	190.56 (30.77)	22.68 (-41.39–86.76)	0.49	0.50
M72L rs696217	Dominant	C/C (n = 667)	51.69 (0.51)	0.00			C/C (n = 667)	168.87 (3.86)	0.00		
M72L rs696217	Dominant	C/A-A/A (n = 116)	53.01 (1.20)	1.32 (-1.27–3.91)	0.32	0.45	C/A-A/A (n = 116)	164.05 (7.79)	-4.82 (-235.36–146.94)	0.62	0.67
L90G rs4684677	-	A/A (n = 743)	51.88 (0.48)	0.00			A/A (n = 737)	169.10 (3.62)	0.00		
L90G rs4684677	-	A/T (n = 60)	51.02 (1.70)	-0.87 (-4.35–2.62)	0.63	0.31	A/T (n = 59)	171.32 (12.33)	2.22 (-23.74–28.19)	0.87	0.85

*Adjusted for age, gender, dyslipemic treatment and BMI.

BMI: body mass index. N: number; SD: standard deviation.

No association was found between MS according to IDF criteria and ghrelin polymorphisms. However, carriers of the A/A genotype in -501AC polymorphism presented a lower number of MS components in comparison to A/C+C/C: 2.43 (0.07) vs 2.63 (0.06), difference in mean 0.19 (0.02–0.37), p = 0.031, after adjusting for age and gender, p = 0.062.

### Analysis by gender

In men, the R51Q polymorphism was associated with waist circumference. A/G genotype was associated with higher waist circumference (A/G: 108 (2.68) cm vs G/G 100.63 (0.48) cm, difference in mean (CI: 95%), adjusted for age and BMI 5.57 (0.18–10.96) cm, p = 0.043) (Table 5). The -604GA and M72L polymorphisms were associated with COB by IDF criteria. Carriers of the A/A genotype in -604GA presented a higher risk of COB (OR: 2.27 (1.11–4.54); p = 0.020 after adjusting for age and BMI) and carriers of the A/C genotype in M72L presented a higher risk of COB (OR: 3.03 (1.10–8.40); p = 0.023 after adjusting for age and BMI) (Table 5). No association was found with lipid profile, glucose levels, diabetes or hypertension. Also -604GA and M72L polymorphisms were associated with metabolic syndrome according to IDF criteria. Carriers of A/A genotype in -604GA presented higher risk of MS (OR: 2.32 (1.021–5.26); p = 0.038 after adjusting for age and BMI) and carriers of A/C genotype in M72L presented higher risk of MS (OR: 5.27 (1.54–18.03); p = 0.004 after adjusting for age and BMI).

**Table 5:** Ghrelin polymorphism associations in men.

SNPs	Model	Genotype (N)	Waist (cm) Mean (SD)	Difference (95% CI) (cm)	P-value	P-value adjusted*	CENTRAL OBESITY COB (%)	CENTRAL OBESITY No COB (%)	OR (95% CI)	P-value	P-value adjusted*
-994CT rs26312	Dominant	C/C (n = 321)	100.53 (0.50)	0.00			250 (80.1)	71 (82.6)	1.00		
-994CT rs26312	Dominant	C/T- T/T (n = 77)	101.61 (1.28)	1.08 (-1.28–3.45)	0.37	0.51	62 (19.9)	15 (17.4)	1.17 (0.63–2.19)	0.61	0.65
-604GA rs27647	Dominant	A/A (n = 122)	101.05 (0.85)	0.00			103(33.0)	19 (22.1)	1.00		
-604GA rs27647	Dominant	G/A- G/G (n = 276)	100.61 (0.58)	-0.44 (-2.46–1.59)	0.67	0.28	209 (67.0)	67 (77.9)	**0.58 (0.33–1.01) 0.44 (0.22–090)** *	**0.047**	**0.02**
-501AC rs26802	Dominant	A/A- (n = 179)	100.44 (0.70)	0.00			140 (45.0)	39 (45.4)	1.00		
-501AC rs26802	Dominant	A/C-C/C (n = 218)	101.01(0.65)	0.58 (-1.30–2.46)	0.55	0.70	171 (55.0)	47 (54.6)	1.01 (0.63–1.84)	0.96	0.31
R51Q	-	G/G (n = 385)	100.63 (0.48)	0.00			303 (98.4)	86 (100)	1.00		
R51Q	-	G/A (n = 5)	108.00 (2.68)	7.37 (-1.01–15.75) **5.57 (0.18–10.96)** *	0.086	**0.043**	5 (1.6)	0 (0.0)	NA	0.12	0.16
M72L rs696217	Dominant	C/C (n = 343)	100.59 (0.50)	0.00			264 (85.4)	79 (91.9)	1.00		
M72L rs696217	Dominant	C/A (n = 52)	101.77 (1.60)	1.18 (-1.60–3.96)	0.41	0.12	45 (14.6)	7 (8.1)	1.95 (0.83–4.43) **3.03 (1.10–8.40)** *	0.10	**0.023**
L90G rs4684677	-	A/A (n = 368)	100.61 (0.50)	0.00			288 (92.0)	80 (93.0)	1.00		
L90G rs4684677	-	A/T (n = 31)	102.39 (1.57)	1.78 (-1.70–5.25)	0.32	0.41	25 (8.0)	6 (7.0)	1.16 (0.46–2.92)	0.75	0.87

*Adjusted for age and BMI.

NA: not available. BMI: body mass index. N: number; SD: standard deviation.

In women, the -501AC polymorphism was associated with being overweight, this being more prevalent in C/C carriers compared to A/A-A/C (OR: 2.31 (1.14–4.65), p = 0.015), after adjusting for age OR: 2.28 (1.13–4.62), p = 0.017). The L90G and -501AC polymorphisms were associated with waist circumference (Table 6). The A/A genotype in L90G was associated with higher waist circumference (A/A: 96.08 (0.63) cm vs A/T 90.93 (2.86) cm; difference in mean adjusted for age and BMI: 4.21 (0.79–7.63) cm, p = 0.016). The C/C genotype in 501AC was associated with higher waist circumference (C/C: 99.21 (2.07) vs A/A+A/C 95.17 (0.65) cm, difference adjusted for age and BMI 3.59 (0.77–6.40), p = 0.013). No significant association was found with COB. Total cholesterol and LDL-cholesterol were associated with -604GA and -501AC polymorphisms (Table 6). Carriers of A/A genotype in -604GA presented lower levels of total cholesterol (A/A: 210.98 (2.87) vs A/G-G/G 220.77 (2.42), difference in mean (CI: 95%) 9.79 (1.71–17.87), p = 0.018, adjusted for age and BMI, p = 0.082) and lower levels of LDL-cholesterol (A/A: 123.47 (2.77) vs A/G-G/G 132.08 (2.12), difference in mean (CI: 95%) 8.60 (1.36–15.84), p = 0.020, adjusted for age and BMI, p = 0.049). Carriers of the C/C genotype in -501AC presented lower levels of total cholesterol (C/C: 207.75 (4.57) vs A/A-A/C 219.09 (2.06), difference in mean (CI: 95%) 11.34 (0.49–23.16), p = 0.061, adjusted for age and BMI, p = 0.074) and lower levels of LDL-cholesterol (C/C: 121.66 (4.36) vs A/A-A/C 130.52 (1.84), difference in mean (CI: 95%) 8.76 (1.75–19.47) cm, p = 0.10, adjusted for age and BMI, p = 0.035). No association was found with glucose levels, diabetes, hypertension or MS.

**Table 6:** Ghrelin polymorphisms association in women.

**SNPs**	**Model**	**Genotype (N)**	**Waist (cm) Mean (SD)**	**Difference (95% CI) (cm)**	**P-value**	**P-value adjusted** *	**CENTRAL OBESITY COB (%)**	**CENTRAL OBESITY No COB (%)**	**OR (95% CI)**	**P-value**	**P-value adjusted** *
-994CT rs26312	Dominant	C/C (n = 298)	96.26 (0.72)	0.00			268 (77.9)	31 (79.5)	1.00		
-994CT rs26312	Dominant	C/T- T/T (n = 84)	93.75 (1.23)	-2.51 (-5.46–0.43)	0.095	0.14	76 (22.1)	8 (20.5)	1.10 (0.49–2.49)	0.82	0.71
-604GA rs27647	Dominant	A/A (n = 115)	96.59 (1.19)	0.00			104(30.4)	11 (27.5)	1.00		
-604GA rs27647	Dominant	G/A- G/G (n = 266)	97.17 (0.73)	-1.42 (-4.10–1.26)	0.30	0.34	238 (69.6)	29 (72.5)	0.87 (0.42–1.80)	0.70	0.73
-501AC rs26802	Recessive	A/A-A/C- (n = 340)	95.17 (0.65)	0.00			116 (33.8)	17 (42.5)	1.00		
-501AC rs26802	Recessive	C/C (n = 42)	99.21 (2.07)	**4.05 (0.15–7.95) 3.59(0.77–6.40)** *	**0.043**	**0.013**	227 (66.2)	23 (57.5)	1.45 (0.74–2.81) **1.30(0.57–2.95)** *	0.28	0.05
R51Q	-	G/G (n = 371)	95.58 (0.63)	0.00			332 (99.1)	39 (97.5)	1.00		
R51Q	-	G/A (n = 4)	87.00 (3.87)	-8.58 (-20.60–3.44)	0.16	0.16	3 (0.9)	1 (2.5)	0.35 (0.04–3.47)	0.42	0.26
M72L rs696217	Dominant	C/C (n = 311)	95.41 (0.72)	0.00			274 (81.8)	37 (92.5)	1.00		
M72L rs696217	Dominant	C/A-A/A (n = 64)	95.88 (1.24)	0.47 (-2.82–3.75)	0.78	0.99	61 (18.2)	3 (7.5)	2.75 (0.82–9.20) **3.60(0.83–15.58)** *	0.063	**0.056**
L90G rs4684677	-	A/A (n = 355)	96.08 (0.63)	0.00			323 (93.6)	33 (84.6)	1.00		
L90G rs4684677	-	A/T (n = 28)	90.93 (2.86)	-**5.15 (-9.83–0.48) -4.21 (-7.63–0.79)** *	**0.031**	**0.016**	22 (6.4)	6 (15.4)	**0.37 (0.14–0.99)** 0.27(0.07–1.10)*	**0.066**	0.079
**SNPs**	**Model**	**Genotype (N)**	**TC (mg/dL) Mean (SD)**	**Difference (95% CI) (cm)**	**P-value**	**P-value adjusted** *	**Genotype (N)**	**LDL-C (mg/dL) Mean (SD)**	**Difference (95% CI) (cm)**	**P-value**	**P-value adjusted** *
-994CT rs26312	Dominant	C/C (n = 309)	218.03 (2.16)	0.00			C/C (n = 309)	129.94 (1.91)	0.00		
-994CT rs26312	Dominant	C/T- T/T (n = 86)	217.48 (4.03)	-0.56 (-9.60–8.49)	0.90	0.84	C/T- T/T (n = 86)	127.94 (3.75)	-1.99 (-10.09–6.10)	0.63	0.90
-604GA rs27647	Dominant	A/A (n = 120)	210.98 (2.87)	0.00			A/A (n = 120)	123.47 (2.77)	0.00		
-604GA rs27647	Dominant	G/A- G/G (n = 274)	220.77 (2.42)	**9.79 (1.71–17.87)** 9.17 (-1.10–19.45)**#**	**0.018**	0.082	G/A- G/G (n = 274)	132.08 (2.12)	**8.60 (1.36–15.84) 9.26(0.09–18.43)** **#**	**0.02**	**0.049**
-501AC rs26802	Recessive	A/A-A/C- (n = 351)	219.09 (2.06)	0.00			A/A-A/C- (n = 351)	130.52 (1.84)	0.00		
-501AC rs26802	Recessive	C/C (n = 44)	207.75 (4.57)	-11.34 (-23.16–0.49) -14.94 (-31.26–1.38)**#**	**0.061**	0.074	C/C (n = 44)	121.66 (4.36)	-8.86 (-19.47–1.75) **-15.71 (-30.26–1.16)** **#**	0.1	**0.035**
R51Q	-	G/G (n = 383)	217.36 (1.94)	0.00			G/G (n = 383)	129.29 (1.74)	0.00		
R51Q	-	G/A (n = 4)	212.75 (7.65)	-4.61 (-41.95–32.72)	0.81	0.60	G/A (n = 4)	118.25 (12.32)	-11.04 (-44.60–22.52)	0.52	0.47
M72L rs696217	Dominant	C/C (n = 322)	217.57 (2.10)	0.00			C/C (n = 322)	129.35 (1.88)	0.00		
M72L rs696217	Dominant	C/A- A/A (n = 65)	216.03 (4.88)	-1.54 (-11.64–8.56)	0.76	0.82	C/A-A/A (n = 65)	128.29 (4.44)	-1.06 (-10.15–8.02)	0.74	0.73
L90G rs4684677	-	A/A (n = 367)	217.60 (1.96)	0.00			A/A (n = 367)	128.91 (1.76)	0.00		
L90G rs4684677	-	A/T (n = 29)	218.48 (7.26)	0.88 (-13.36–15.11)	0.90	0.94	A/T (n = 29)	134.21 (6.33)	5.30 (-7.44–18.04)	0.82	0.80

*Adjusted for age and BMI.

^#^Adjusted by age, dyslipemic treatment and BMI.

BMI: body mass index. N: number; SD: standard deviation.

## Discussion

The association between ghrelin polymorphisms and MS components has been a matter of interest in recent years. However, little is known about this relationship in the elderly population. In the present study, we investigated whether ghrelin gene polymorphisms were related to MS components in elderly Caucasian subjects participating in the Mataró and the Río Hortega Studies, both conducted in Spain [22,23]. We did not find any relationship between fasting total plasma ghrelin concentrations and the six SNPs studied; similar results have also been reported by other authors [28,29] in relation to total and acetylated ghrelin concentrations and M72L and L90G. Ukkola [30] found lower levels of ghrelin in Arg51Gln subjects. Unfortunately, we did not measure acetylated ghrelin, which is known to be the accepted active ghrelin form of the peptide responsible for its cardiometabolic actions. Functional consequences of the variants of the six SNPs are still unknown; variants of -604CT (located in the promoter region of the ghrelin gene) and M72L (between mature ghrelin and obestatin) are not associated with changes in mature ghrelin, but these variants may modulate protein processing or mRNA stability, and may modify secretion or activity of ghrelin. L90G variants have been related to changes in the obestatin peptide, but its possible consequences have not been elucidated. Recent studies have found that -994CT [30], as well as M72L and -604CT influence the expression of the ghrelin gene, but L90G does not [17].

We found an association between the -604GA polymorphism with COB, total and LDL-cholesterol in all subjects. In men, we found an association with COB and MS (IDF criteria), while in women it was associated only with total and LDL-cholesterol. Polymorphism -501AC was associated with BMI, LDL-cholesterol and triglycerides in all subjects. In women, -501AC was associated with waist circumference, overweight, total cholesterol and LDL-cholesterol.

R51Q presented an association with being overweight in all subjects and was associated with waist circumference in men. M72L was associated with COB in all subjects and in men, it was associated with MS. L90G was associated with waist circumference in women. Haplotype analysis also confirmed the influence of ghrelin polymorphisms in some MS components, particularly waist circumference and COB, in which SNPs haplotype 4 showed a strong risk effect. All these data may indicate the importance of ghrelin polymorphisms in anthropometric data and with parameters related to lipid profile while there is no influence on other parameters such as glucose metabolism or blood pressure. In addition, these associations are influenced by gender as some of them are only present in men or in women. In any case, these results should be interpreted cautiously due to the size of our sample study and their low frequency of appearance.

Other authors have found different results when evaluating MS components and ghrelin SNPs and little data is available in relation to -501AC ghrelin SNP. Hubacek et al [31] studied the association between this SNP and BMI but no significant association was found. Other SNPs have been studied in relation to obesity and BMI, especially M72L, some with a positive association [29,32,33] and others with no relationship [34]. In our male sample, A/C genotype in M72L presented higher prevalence of COB and in our female sample, A/A genotype in L90G was associated with higher waist circumference. Similar data have been reported in relation to lipid profile, where M72L has been associated with differences in HDL [16,35] and triglycerides [35] and no association with lipid profile has been reported for -501AC [35,36] or R51Q [35].

Several studies have found a protector role of M72L for diabetes [37–39], which can be associated with an effect on insulin resistance, increasing its sensibility [17,32], while others have found no association [40,41]. In our sample, we have not found any association with diabetes or glucose levels.

No association between the five studied SNPS and hypertension was found in our subjects. However other reports have described significant associations with -604G/A, -501 A/C, L90G and M72L [42] and -501 A/C [37].

In relation to MS, carriers of the A/A genotype in -604GA and the A/C genotype in M72L presented a higher prevalence of COB and MS according to IDF criteria, but this association was only observed in men. Contradictory results have been previously reported: for example an association between M72L and MS [16,21] and no association [19,43].

Our study has several limitations. It is a population-based study where non-institutionalized individuals were selected. However, if we had included younger subjects we would have avoided the over-representation of certain genotypes because of survival bias. The amount of subjects that were finally included was also restricted to the recruitment possibilities in the specific geographical areas involved in the study; a stronger statistical power would have probably been possible with a higher number of participants, especially in low frequency polymorphisms. Moreover, the measurement of acetylated and desacetylated ghrelin rather than total ghrelin, as well as obestatin, would have helped to explore potential relationships not found with our current design. Finally, the high prevalence of COB and MS according to IDF criteria in our subjects may have also contributed to a bias [44].

In conclusion, in our cohort of aged Spanish-dwelling individuals, Ghrelin gene variants are associated with certain components of MS, in particular with central obesity and hypertension.

## Supporting Information

S1 Table Prevalence of the genotypes in whole sample and by gender. (DOCX)

S2 Table Linkage disequilibrium of the polymorphisms analyzed in our population measured by D’ statistic. (DOCX)
