# Supplementary material for: Ghrelin Gene Variants Influence on Metabolic Syndrome Components in Aged Spanish Population
Source: PLoS One. 2015 Sep 16;10(9):e0136931. doi: 10.1371/journal.pone.0136931 (PMC4573319; doi:10.1371/journal.pone.0136931)
Supplement: S1 Table — (DOCX) [file pone.0136931.s001.docx]

**Supporting Information Captions**

S1 Table. Prevalence of the genotypes in whole sample and by gender.

| SNPs Pol. | Genotypes  All sample | | | Genotypes  Women | | | Genotypes  Men | |  | |  |
| --- | --- | --- | --- | --- | --- | --- | --- | --- | --- | --- | --- |
| -994CT  rs 26312 | C/C  80% | C/T  19 % | T/T  1% | C/C  78% | C/T  21% | T/T  1% | C/C  81% | C/T  18% | |  | T/T  1% |
| -604GA  rs27647 | A/A  30% | G/A  54% | G/G  16% | A/A  30% | G/A  56% | G/G  14% | A/A  31% | G/A  52% | |  | G/G  17% |
| -501AC  rs 26802 | A/A  41% | C/A  49% | C/C  10% | A/A  36% | C/A  53% | C/C  11% | A/A  45% | C/A  46% | |  | C/C  9% |
| R51Q  rs34911341 | G/G  99% | G/A  1% | - | G/G  99% | G/A  1% | - | G/G  99% | G/A  1% | |  | - |
| M72L  rs696217 | C/C  85% | C/A  15% | - | C/C  84% | C/A  16% | - | C/C  87% | C/A  13% | |  |  |
| L90G  rs4684677 | A/A  93% | A/T  7% | - | A/A  93% | A/T  7% | - | A/A  92% | A/T  8% | |  |  |
