# Supplementary material for: Ghrelin Gene Variants Influence on Metabolic Syndrome Components in Aged Spanish Population
Source: PLoS One. 2015 Sep 16;10(9):e0136931. doi: 10.1371/journal.pone.0136931 (PMC4573319; doi:10.1371/journal.pone.0136931)
Supplement: S2 Table — (DOCX) [file pone.0136931.s002.docx]

**Supporting Information Captions**

S2 Table. Linkage disequilibrium of the polymorphisms analyzed in our population measured by D’ statistic.

| D' statistic | -994CT | -604GA | -501AC | R51Q | M72L | L90G |
| --- | --- | --- | --- | --- | --- | --- |
| -994CT |  | 0.9129 | 0.3785 | 0.8753 | 0.9022 | 0.3013 |
| -604GA |  |  | 0.8342 | 0.7355 | 0.9985 | 0.895 |
| -501AC |  |  |  | 0.9623 | 0.2683 | 0.8504 |
| R51Q |  |  |  |  | 0.2201 | 0.1605 |
| M72L |  |  |  |  |  | 0.1747 |
| L90G |  |  |  |  |  |  |
